# INTEGRATING THE GERIATRIC 5MS INTO MOVEMENT ANALYSIS AND COLLABORATIVE GERIATRIC PHYSICAL THERAPY

**DOI:** 10.1093/geroni/igae098.2849

**Published:** 2024-12-31

**Authors:** Gregory Hartley, Kenneth Miller, Myla Quiben, Marni Larkin, Michelle Lusardi, Susan Leach, Laura Gras

**Affiliations:** University of Miami Miller School of Medicine, Coral Gables, Florida, United States; Medical University of South Carolina, Charleston, South Carolina, United States; University of North Texas Health Science Center, Fort Worth, Texas, United States; Gyrotonic Manhasset Physical Therapy, Manhasset, New York, United States; Sacred Heart University, Fairfield, Connecticut, United States; University of New Mexico, Albuquerque, New Mexico, United States; Ithaca College, Ithaca, New York, United States

## Abstract

The Geriatric 5Ms (Matters Most, Medications, Mind, Mobility, Multicomplexity) address complexity of health care for aging adults. The 5Ms are important for physical therapists (PT); members of the health care team concerned with musculoskeletal pain, mobility, and functional capacity. The Academy of Geriatric Physical Therapy, a component of the American Physical Therapy Association, was charged with incorporating emerging movement system science and geriatric care models into a comprehensive PT assessment framework for primary care and initial PT patient encounters. The product, a geriatric movement system assessment framework, incorporates principles of multiple models including the International Classification of Functioning Disability and Health, and other movement-based patient/client management models. Observational movement analysis of key tasks required for safe independent function using the 5M model helps detect needed resources and constraints. Results of movement analysis from the patient examination informed by the 5Ms, impact selection of tests and measures for further analysis of baseline functions. Results allow clinicians to set priorities for targeted interventions addressing body structure/function, activity, and/or participation, and assess outcomes of care. The theoretical basis for this novel approach to movement assessment in aging adults is discussed. Case studies illustrate application of the framework with attention to understanding patient resources as part of intervention strategies. Adoption of the 5Ms into geriatric physical therapy highlights essential geriatric principles and bridges practice across interprofessional teams.

